# Crystal Structure, Chemical Bonding and Magnetism Studies for Three Quinary Polar Intermetallic Compounds in the (Eu_1−*x*_Ca*_x_*)_9_In_8_(Ge_1−*y*_Sn*_y_*)_8_ (*x* = 0.66, *y* = 0.03) and the (Eu_1−*x*_Ca*_x_*)_3_In(Ge_3−*y*_Sn_1+*y*_) (*x* = 0.66, 0.68; *y* = 0.13, 0.27) Phases

**DOI:** 10.3390/ijms16049017

**Published:** 2015-04-22

**Authors:** Hyein Woo, Eunyoung Jang, Jin Kim, Yunho Lee, Jongsik Kim, Tae-Soo You

**Affiliations:** 1 Department of Chemistry, Chungbuk National University, Cheongju, Chungbuk 362-763, Korea; E-Mails: hiwoo52@chungbuk.ac.kr (H.W.); s2jeyoung@chungbuk.ac.kr (E.J.); 2 Department of Chemistry, Korea Advanced Institute of Science and Technology, Daejeon 305-701, Korea; E-Mails: kj0815@kaist.ac.kr (J.K.); yunholee@kaist.ac.kr (Y.L.); 3 Department of Chemistry, Dong-A University, Busan 604-714, Korea; E-Mail: jskimm@dau.ac.kr

**Keywords:** polar intermetallics, electronic structure, chemical bonding, single-crystal X-ray diffraction, magnetism

## Abstract

Three quinary polar intermetallic compounds in the (Eu_1−*x*_Ca*_x_*)_9_In_8_(Ge_1−*y*_Sn*_y_*)_8_ (*x* = 0.66, *y* = 0.03) and the (Eu_1−*x*_Ca*_x_*)_3_In(Ge_3-*y*_Sn_1+*y*_) (*x* = 0.66, 0.68; *y* = 0.13, 0.27) phases have been synthesized using the molten In-metal flux method, and the crystal structures are characterized by powder and single-crystal X-ray diffractions. Two orthorhombic structural types can be viewed as an assembly of polyanionic frameworks consisting of the In(Ge/Sn)_4_ tetrahedral chains, the bridging Ge_2_ dimers, either the annulene-like “12-membered rings” for the (Eu_1−*x*_Ca*_x_*)_9_In_8_(Ge_1−*y*_Sn*_y_*)_8_ series or the *cis-trans* Ge/Sn-chains for the (Eu_1−*x*_Ca*_x_*)_3_In(Ge_3−*y*_Sn_1+*y*_) series, and several Eu/Ca-mixed cations. The most noticeable difference between two structural types is the amount and the location of the Sn-substitution for Ge: only a partial substitution (11%) occurs at the In(Ge/Sn)_4_ tetrahedron in the (Eu_1−*x*_Ca*_x_*)_9_In_8_(Ge_1−*y*_Sn*_y_*)_8_ series, whereas both a complete and a partial substitution (up to 27%) are observed, respectively, at the *cis-trans* Ge/Sn-chain and at the In(Ge/Sn)_4_ tetrahedron in the (Eu_1−*x*_Ca*_x_*)_3_In(Ge_3−*y*_Sn_1+*y*_) series. A series of tight-binding linear muffin-tin orbital calculations is conducted to understand overall electronic structures and chemical bonding among components. Magnetic susceptibility measurement indicates a ferromagnetic ordering of Eu atoms below 5 K for Eu_1.02(1)_Ca_1.98_InGe_2.87(1)_Sn_1.13_.

## 1. Introduction

Polar intermetallic and Zintl phase compounds have been one of the most interesting research topics for solid-state chemists to investigate the co-relationship among composition-structure-property and to apply the obtained knowledge to various energy-related materials, such as thermoelectrics, magnetocalorics and magnetoresistance materials [1,2,3,4,5,6]. Among these compounds, the rare-earth metal containing compounds are worth exploring due to their intriguing chemical and physical characteristics derived by electrons in the 4*f* orbitals [7,8,9,10,11]. In particular, some polar intermetallics containing europium with the half-filled 4*f* orbitals are known to show anomalous magnetic properties [12,13,14].

Our group has continuously investigated rare-earth metal containing polar intermetallics as well as Zintl phase compounds and successfully synthesized several novel compounds as reported in recent articles [14,15,16,17,18,19,20]. As a part of our ongoing systematic investigations for the Eu-containing polar intermetallic compounds, we attempted to expand the variety of the recently reported (Eu_1−*x*_Ca*_x_*)_9_In_8_Ge_8_ series by substituting elements and adjusting reaction conditions. The series of reaction attempts eventually revealed that the heavier Sn successfully replaced Ge in this series resulting in producing three title compounds: Eu_3.04(4)_Ca_5.96_In_8_Ge_7.77(2)_Sn_0.23_, Eu_1.02(1)_Ca_1.98_InGe_2.87(1)_Sn_1.13_ and Eu_0.95(1)_Ca_2.05_InGe_2.73(1)_Sn_1.27_, which were the first three quinary derivatives from the (Eu_1−*x*_Ca*_x_*)_9_In_8_Ge_8_ and the (Eu_1−*x*_Ca*_x_*)_3_In_2_Ge_3_ series, respectively [12,14]. Interestingly, the amount and the location of the Sn-substitution for Ge differentiated two structural types: only a partial Sn substitution for Ge at the In(Ge/Sn)_4_ tetrahedron resulted in the (Eu_1__−_*_x_*Ca*_x_*)_9_In_8_(Ge_1__−_*_y_*Sn*_y_*)_8_ series, whereas a partial and a complete Sn substitutions for Ge in the polyanionic framework produced the (Eu_1−*x*_Ca*_x_*)_3_In(Ge_3−*y*_Sn_1+*y*_) series.

In this article, we report crystal structures of two title phases in terms of the amount and the location of the Sn-substitution and provide the rationale for these phenomena using the atomic size-factor perspective and comprehensive theoretical analyses. Tight-binding linear muffin-tin orbital (TB-LMTO) calculations [21,22,23,24,25] were conducted using two structural models with idealized compositions. Density of states (DOS) and crystal orbital Hamilton population (COHP) curves [26] were thoroughly studied to understand orbital contributions for particular energy regions and chemical bonding from interatomic interactions. Physical and chemical analyses including energy-dispersive X-ray spectroscopy (EDS), scanning electron microscope (SEM), differential scanning calorimeter (DSC), and magnetization were also performed.

## 2. Results and Discussion

### 2.1. Crystal Structure Descriptions

#### 2.1.1. (Eu_1−*x*_Ca*_x_*)_9_In_8_(Ge_1−*y*_Sn*_y_*)_8_ Phase

**Figure 1:**
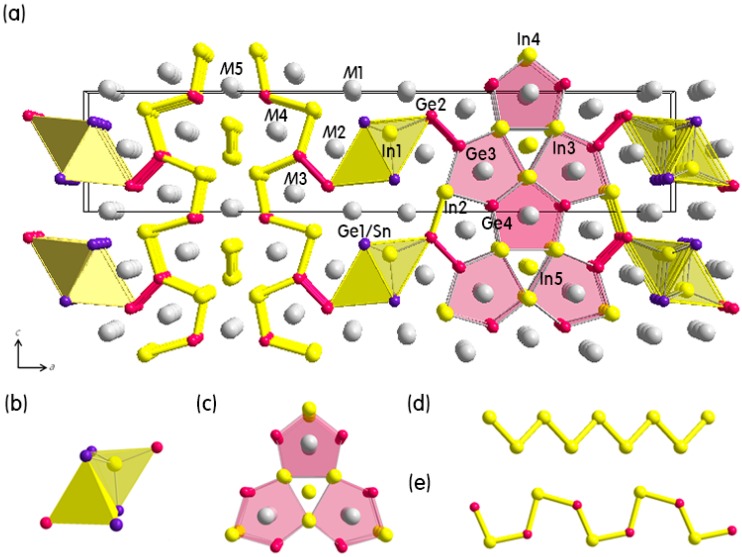
(**a**) Combined ball-and-stick and polyhedral representation for the crystal structure of the (Eu_1−*x*_Ca*_x_*)_9_In_8_(Ge_1−*y*_Sn*_y_*)_8_ (*x* = 0.66, *y* = 0.03) series viewed down along the *b-*axis; (**b**) The edge-sharing In(Ge/Sn)_4_ tetrahedra; (**c**) The “12-membered rings”; (**d**) The 1*D zig-zag* In-chain; and (**e**) The 1*D cis-trans* Ge/In-chain are also illustrated. Unit cell is outlined in black. Color codes are as follows: *M*(Eu/Ca-mixed site), gray; Ge, magenta; Ge1/Sn-mixed site, purple; and In, yellow.

**Figure 2:**
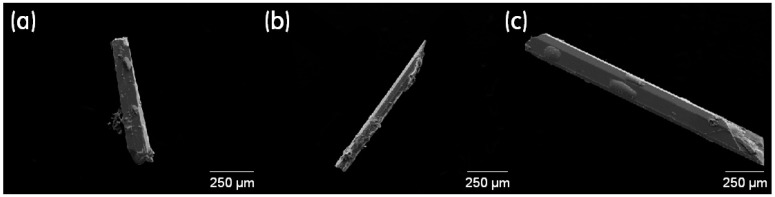
SEM images for bar-/needle-shaped single-crystals of (**a**) Eu_3.04(4)_Ca_5.96_In_8_Ge_7.77(2)_Sn_0.23_; (**b**) Eu_1.02(1)_Ca_1.98_InGe_2.87(1)_Sn_1.13_; and (**c**) Eu_0.95(1)_Ca_2.05_InGe_2.73(1)_Sn_1.27_. Small amounts of In-flux metals remaining on the surface of single-crystals are also observed. Scale bar = 250 μm.

Eu_3.04(4)_Ca_5.96_In_8_Ge_7.77(2)_Sn_0.23_ is the first quinary derivative of the recently reported (Eu_1−*x*_Ca*_x_*)_9_In_8_Ge_8_ [14] series having a partial Sn-substitution for Ge. The title compound adopted its parental Eu_2.94(2)_Ca_6.06_In_8_Ge_8_-type structure and crystallized in the orthorhombic *Pmmn* space group (*Z* = 2, Pearson code *oP*50) with 14 crystallographically independent sites in the asymmetric unit (Table 1). The overall crystal structure is illustrated in Figure 1, and a SEM image of a needle-/bar-shaped single-crystal is also shown in Figure 2a.

**Table 1:** Single-crystal crystallographic data and structure refinement results for Eu_3.04(4)_Ca_5.96_In_8_Ge_7.77(2)_Sn_0.23_, Eu_1.02(1)_Ca_1.98_InGe_2.87(1)_Sn_1.13_ and Eu_0.95(1)_Ca_2.05_InGe_2.73(1)_Sn_1.27_.

Empirical Formula	Eu_3.04(4)_Ca_5.96_In_8_Ge_7.77(2)_Sn_0.23_	Eu_1.02(1)_Ca_1.98_InGe_2.87(1)_Sn_1.13_	Eu_0.95(1)_Ca_2.05_InGe_2.73(1)_Sn_1.27_
fw, g·mol^−1^	2211.05	691.24	690.25
space group, *Z*	*Pmmn* (No. 59), 2	*Pnma* (No. 62), 4	
unit cell dimension, Å	*a =* 36.963(2)	*a* = 7.4974(8)	*a =* 7.5096(3)
	*b* = 4.5176(2)	*b* = 4.4847(4)	*b* = 4.4959(2)
	*c* = 7.5155(3)	*c* = 23.819(3)	*c* = 23.8518(9)
volume, Å^3^	1254.97(9)	800.88(14)	805.29(6)
density (ρ_calcd_), g·cm^−3^	5.851	5.733	5.693
absorption coefficient (μ), cm^−1^	252.39	259.44	252.39
GOF on *F*^2^	1.074	1.057	1.059
*R ^a^* [*I* > 2σ(*I*)]	*R*_1_ = 0.0348	*R*_1_ = 0.0264	*R*_1_ = 0.0256
	*wR*_2_ = 0.0595	*wR*_2_ = 0.0462	*wR*_2_ = 0.0400
*R* [all data]	*R*_1_ = 0.0540	*R*_1_ = 0.0399	*R*_1_ = 0.0411
	*wR*_2_ = 0.0637	*wR*_2_ = 0.0541	*wR*_2_ = 0.0472
largest diff. peak and hole, e^−^·Å^−3^	2.423 and −2.527	1.180 and −1.821	1.859 and −1.585

*^a^ R*_1_ = Σ||F_o_| − |F_c_||/Σ|F_o_|; *wR*_2_ = [Σ[*w*(*F*_o_^2^ − *F*_c_^2^]/Σ[*w*(*F*_o_^2^)^2^]]^1/2^, where *w* = 1/[σ^2^*F*_o_^2^ + (A∙*P*)^2^ + B∙*P*], and *P* = (*F*_o_^2^ + 2*F*_c_^2^)/3; *A* and *B* are weight coefficients.

The overall crystal structure can be viewed as an assembly of the three-dimensional (3*D*) polyanionic framework consisting of three types of anions and five mixed-cationic sites embedded within the framework. Furthermore, the 3*D* framework can be understood as a combination of (1) the one-dimensional (1*D*) In(Ge/Sn)_4_ tetrahedral chains extending along the *b*-axis direction and (2) the distorted annulene-like “12-membered rings”, which stacked on top of each other along the *b*-axis direction and eventually resulted in forming three edge-sharing pentagonal-prisms (See the right side of Figure 1). This anionic 12-membered ring can alternately be viewed as a combination of the 1*D zig-zag* In-chains (Figure 1d) and the 1*D cis-trans* Ge/In-chains (Figure 1e) propagating, respectively, along the *b*-axis and the *c*-axis as illustrated in the left side of Figure 1. According to this alternative perspective, the structure type of the (Eu_1−*x*_Ca*_x_*)_9_In_8_(Ge_1−*y*_Sn*_y_*)_8_ phase can more easily be comparable to the other title phase, the (Eu_1−*x*_Ca*_x_*)_3_In(Ge_3−*y*_Sn_1+*y*_) series, which will be discussed in a subsequent section. In addition, the In(Ge/Sn)_4_ chains and the 12-membered rings are further connected via the Ge_2_ dimers along the *a*-axis direction. Interestingly, the newly introduced Sn partially substituted for Ge (11%) only at the Ge1 site in the In(Ge/Sn)_4_ tetrahedron among total nine anionic sites. The rest of Ge and In sites showed no sign of the Sn-substitution. The limited Sn-substitution at the Ge1 site is clearly distinctive from the Sn-substitution occurred in the (Eu_1−*x*_Ca*_x_*)_3_In(Ge_3−*y*_Sn_1+*y*_) series. We will further discuss about the different Sn-substitutions in the subsequent section.

The overall crystal structure can be viewed as an assembly of the three-dimensional (3*D*) polyanionic framework consisting of three types of anions and five mixed-cationic sites embedded within the framework. Furthermore, the 3*D* framework can be understood as a combination of (1) the one-dimensional (1*D*) In(Ge/Sn)_4_ tetrahedral chains extending along the *b*-axis direction and (2) the distorted annulene-like “12-membered rings”, which stacked on top of each other along the *b*-axis direction and eventually resulted in forming three edge-sharing pentagonal-prisms (See the right side of Figure 1). This anionic 12-membered ring can alternately be viewed as a combination of the 1*D zig-zag* In-chains (Figure 1d) and the 1*D cis-trans* Ge/In-chains (Figure 1e) propagating, respectively, along the *b*-axis and the *c*-axis as illustrated in the left side of Figure 1. According to this alternative perspective, the structure type of the (Eu_1−*x*_Ca*_x_*)_9_In_8_(Ge_1−*y*_Sn*_y_*)_8_ phase can more easily be comparable to the other title phase, the (Eu_1−*x*_Ca*_x_*)_3_In(Ge_3−*y*_Sn_1+*y*_) series, which will be discussed in a subsequent section. In addition, the In(Ge/Sn)_4_ chains and the 12-membered rings are further connected via the Ge_2_ dimers along the *a*-axis direction. Interestingly, the newly introduced Sn partially substituted for Ge (11%) only at the Ge1 site in the In(Ge/Sn)_4_ tetrahedron among total nine anionic sites. The rest of Ge and In sites showed no sign of the Sn-substitution. The limited Sn-substitution at the Ge1 site is clearly distinctive from the Sn-substitution occurred in the (Eu_1−*x*_Ca*_x_*)_3_In(Ge_3−*y*_Sn_1+*y*_) series. We will further discuss about the different Sn-substitutions in the subsequent section.

The similar types of edge-sharing In(Ge/Sn)_4_ tetrahedral chains have been frequently reported in several recent articles [12,14,20]. In particular, the observed In1-Ge/Sn bond distances in Eu_3.04(4)_Ca_5.96_In_8_Ge_7.77(2)_Sn_0.23_ are 2.816 and 2.896 Å, which are well comparable to the values reported in some compounds: 2.769–2.909 Å in the *M*_9_In_8_Ge_8_ (*M* = Eu/Ca-, Sr/Ca-mixed sites) [14], 2.802–2.887 Å in the (Eu_1−*x*_Ca*_x_*)_4_In_3_Ge_4_ (0.35 ≤ *x* ≤ 0.70) [12], 2.790–2.869 Å in the (Eu_1−*x*_Ca*_x_*)_3_In_2_Ge_3_ (0.78 ≤ *x* ≤ 0.90) [12], 2.716–2.822 Å in the (Sr_1−*x*_Ca*_x_*)_3_In_2_Ge_4_ (*x* = 0.39, 0.49) series [20], and 2.672–2.877 Å in Sr_1.50_Ca_3.50_In_3_Ge_6_ [20]. It is noteworthy to mention that the size of a unit cell (Table 1) involving the In1-Ge/Sn distances in the title compound is not much expanded as compared to those in the parental (Eu_1−*x*_Ca*_x_*)_9_In_8_Ge_8_ series [14] as well as above mentioned examples in spite of the large size difference between Sn and Ge (*r*_Sn_ = 1.40 Å, *r*_Ge_ = 1.22 Å) [27]. The rationale for this observation can surely be provided by a relatively small amount of the Sn substitution only at the Ge1 site, which is not the case of the other title phase, the (Eu_1−*x*_Ca*_x_*)_3_In(Ge_3−*y*_Sn_1+*y*_) series. The Ge-Ge bond distance of the Ge_2_ dimer is 2.554 Å. This value is also well comparable to those observed in above examples including 2.551–2.567 Å in the *M*_9_In_8_Ge_8_ (*M* = Eu/Ca- or Sr/Ca-mixed sites) [14], 2.54 Å in the (Eu_1−*x*_Ca*_x_*)_4_In_3_Ge_4_ (0.35 ≤ *x* ≤ 0.70) [12], 2.527–2.533 Å in the *M*_3_In_2_Ge_3_ (*M* = Eu/Ca-mixed sites) [12], 2.538–2.622 Å in the (Sr_1−*x*_Ca*_x_*)_3_In_2_Ge_4_ (*x* = 0.39, 0.49) series [20], and 2.528–2.550 Å in Sr_1.50_Ca_3.50_In_3_Ge_6_ [20].

As briefly mentioned earlier, there exist five asymmetric Eu/Ca mixed-sites, which are surrounded by Ge and In. In particular, the *M*1 site having *ca*. 30% of Eu occupation has a total coordination number of 9 and shows the distorted square-pyramidal coordination environment. The rest of four cationic sites having a total coordination number of 10 are surrounded by the pentagonal-prismatic environments: the *M*2 and the *M*3 sites are coordinated by six Ge (or Ge/Sn) and four In atoms, whereas the *M*4 and the *M*5 sites are coordinated by six In and four Ge atoms (Figure 1 and Table 2).

**Table 2:** Atomic coordinates, occupation factors and equivalent isotropic displacement parameters (*U*_eq_*^a^*) from single-crystal structure refinements for Eu_3.04(4)_Ca_5.96_In_8_Ge_7.77(2)_Sn_0.23_, Eu_1.02(1)_Ca_1.98_InGe_2.87(1)_Sn_1.13_ and Eu_0.95(1)_Ca_2.05_InGe_2.73(1)_Sn_1.27_.

Atom	*Wyckoff* Site	Occupation Factor		*x*	*y*	*z*	*U*_eq_*^ a^* (Å^2^)
Eu_3.04(4)_Ca_5.96_In_8_Ge_7.77(2)_Sn_0.23_							
*M*1 *^b^*	4*f*	0.298(4)/0.702		0.0456 (1)	1/4	0.0079(2)	0.0066(5)
*M*2 *^b^*	4*f*	0.506(4)/0.494		0.0755(1)	1/4	0.5209(1)	0.0053(3)
*M*3 *^b^*	4*f*	0.271(4)/0.729		0.1443(1)	1/4	0.1498(2)	0.0055(5)
*M*4 *^b^*	4*f*	0.284(4)/0.716		0.1740(1)	1/4	0.6668(2)	0.0060(5)
*M*5 *^b^*	2*a*	0.325(6)/0.675		1/4	1/4	0.0310(3)	0.0068(6)
In1	4*f*	1		0.5172(1)	1/4	0.6502(1)	0.0052(2)
In2	4*f*	1		0.6110(1)	1/4	0.1632(1)	0.0077(2)
In3	4*f*	1		0.7039(1)	1/4	0.6868(1)	0.0055(2)
In4	2*b*	1		3/4	1/4	0.2854(2)	0.0068(3)
In5	2*a*	1		1/4	1/4	0.4544(2)	0.0074(3)
Ge1/Sn	4*f*	0.887(12)/0.113		0.5251(1)	1/4	0.2668(2)	0.0072(4)
Ge2	4*f*	1		0.5867(1)	1/4	0.7882(2)	0.0050(3)
Ge3	4*f*	1		0.6344(1)	1/4	0.5427(2)	0.0050(3)
Ge4	4*f*	1		0.6898(1)	1/4	0.0519(2)	0.0043(3)
Eu_1.02(1)_Ca_1.98_InGe_2.87(1)_Sn_1.13_							
*M*1 *^b^*	4*c*		0.302(3)/0.698	0.0096(1)	1/4	0.0735(1)	0.0070(3)
*M*2 *^b^*	4*c*		0.483(3)/0.517	0.0273(1)	1/4	0.3814(1)	0.0066(2)
*M*3 *^b^*	4*c*		0.232(3)/0.768	0.1640(1)	1/4	0.2267(1)	0.0080(3)
In1	4*c*		1	0.1497(1)	1/4	0.5283(1)	0.0074(1)
Sn1	4*c*		1	0.1623(1)	1/4	0.8244(1)	0.0096(1)
Ge1	4*c*		1	0.0391(1)	1/4	0.7108(1)	0.0060(2)
Ge2	4*c*		1	0.2833(1)	1/4	0.6371(1)	0.0063(2)
Ge3/Sn2	4*c*		0.871(7)/0.129	0.7643(1)	1/4	0.5375(1)	0.0065(2)
Eu_0.95(1)_Ca_2.05_InGe_2.73(1)_Sn_1.27_							
*M*1 *^b^*	4*c*		0.274(2)/0.726	0.0106(1)	1/4	0.0740(1)	0.0077(2)
*M*2 *^b^*	4*c*		0.453(2)/0.547	0.0264(1)	1/4	0.3811(1)	0.0064(2)
*M*3 *^b^*	4*c*		0.220(2)/0.780	0.1642(1)	1/4	0.2269(1)	0.0070(2)
In1	4*c*		1	0.1505(1)	1/4	0.5287(1)	0.0077(1)
Sn1	4*c*		1	0.1624 (1)	1/4	0.8243(1)	0.0088(1)
Ge1	4*c*		1	0.0395(1)	1/4	0.7108(1)	0.0062(1)
Ge2	4*c*		1	0.2837(1)	1/4	0.6374(1)	0.0064(1)
Ge3/Sn2	4*c*		0.734(7)/0.266	0.7635(1)	1/4	0.5377(1)	0.0067(2)

*^a^ U*_eq_ is defined as one third of the trace of the orthogonalized *U*_ij_ tensor; *^b^ M* is refined as statistical mixture of Eu and Ca.

2.1.2. (Eu_1−*x*_Ca*_x_*)_3_In(Ge_3−*y*_Sn_1+*y*_) Phase

Two quinary derivatives of Eu_1.02(1)_Ca_1.98_InGe_2.87(1)_Sn_1.1.03_ and Eu_0.95(1)_Ca_2.05_InGe_2.73(1)_Sn_1.27_ in the (Eu_1−*x*_Ca*_x_*)_3_In(Ge_3−*y*_Sn_1+*y*_) phase have been serendipitously produced when the reaction condition satisfied two following criteria: (1) the loaded Sn content increased up to equal to or slightly higher than the loaded Ge content and (2) the loaded ratio between Eu/Ca- and Ge/Sn-mixture was 1:1. Interestingly, according to a recent article about the (Eu_1−*x*_Ca*_x_*)_4_In_3_Ge_4_ and the (Eu_1−*x*_Ca*_x_*)_3_In_2_Ge_3_ series [12], either one of two phases could selectively be produced by controlling the loaded ratio between Eu and Ca. For instance, the (Eu_1−*x*_Ca*_x_*)_4_In_3_Ge_4_ phase was produced when the ratio varied between *ca*. 2:1 and 1:2, whereas the (Eu_1−*x*_Ca*_x_*)_3_In_2_Ge_3_ phase was obtained only when the loaded Ca content was at least three times larger than Eu, such as Eu:Ca = 1:3 or 1:9. However, in this study, we revealed that two quinary derivatives from the (Eu_1−*x*_Ca*_x_*)_3_In(Ge_3−*y*_Sn_1+*y*_) phase could possibly be obtained even when the loaded Ca contents were much smaller than those claimed in the earlier report, such as Eu:Ca = 1:1.94 and 1:2.16. In addition, the overall atomic % of Eu in title compounds was different from those in the parental (Eu_1−*x*_Ca*_x_*)_3_In_2_Ge_3_ phase, which could not exceed 20% under any attempted reaction conditions. The rationale behind the limited Eu content was attributed to the fact that packing two different size cations in an ordered manner provided very little energy profit [12]. However, in the title phase, it increased up to 34 atomic % and could be explained by the atomic size-factor: the larger-size Sn atoms substituted the smaller-size Ge atoms not only at the vertex of the In(Ge/Sn)_4_ tetrahedron, but also on the 1*D cis-trans* chain. As a result, an overall size of the unit cell including the 3*D* anionic framework expanded large enough to accommodate extra amounts of Eu.

**Figure 3:**
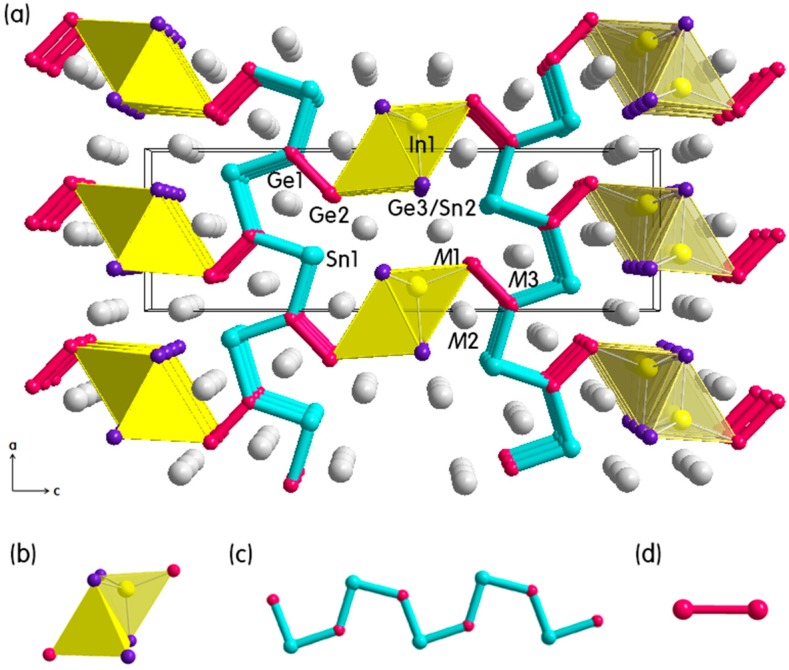
(**a**) Combined ball-and-stick and polyhedral representation for the crystal structure of the (Eu_1−*x*_Ca*_x_*)_3_In(Ge_3−*y*_Sn_1+*y*_) (*x* = 0.66, 0.68; *y* = 0.13, 0.27) phase viewed down along the *b-*axis; (**b**) The edge-sharing In(Ge/Sn)_4_ tetrahedra; (**c**) The 1*D cis*-*trans* Ge/Sn-chains; and (**d**) The Ge_2_ dimers are highlighted in yellow, light-blue, and magenta, respectively. Unit cell is outlined in black, and color codes are as follows: *M*(Eu/Ca-mixed site), gray; Ge, magenta; Ge/Sn mixed-site, purple; In, yellow; and Sn, light blue.

Two title compounds in the (Eu_1−*x*_Ca*_x_*)_3_In(Ge_3−*y*_Sn_1+*y*_) phase adopted the orthorhombic space group *Pnma* (*Z* = 4, Pearson code *oP*32) and contained eight crystallographically independent atomic sites in the asymmetric unit as provided in Table 1 and Table 2. The overall crystal structure can be viewed as an assembly of three structural moieties: (1) the 1*D* edge-sharing In(Ge/Sn)_4_ tetrahedral chains propagating along the *b*-axis direction; (2) the infinite *cis-trans* Ge/Sn-chains extending along the *a*-axis direction and (3) the Ge_2_ dimers bridging these two structural moieties as illustrated in Figure 3. SEM images of two needle-/bar-shaped single-crystals are also displayed in Figure 2b,c. The In(Ge/Sn)_4_ tetrahedral chain resembles the chain observed in Eu_3.04(4)_Ca_5.96_In_8_Ge_7.77(2)_Sn_0.23_ including a partial Sn substitution for Ge, and the In-Ge/Sn bond distances in the tetrahedron (2.779 and 2.914 Å) are well comparable to those in Eu_3.04(4)_Ca_5.96_In_8_Ge_7.77(2)_Sn_0.23_ as well as in some compounds discussed earlier. In particular, the infinite *cis*-*trans* Ge/Sn-chains, in which Ge and Sn atoms were alternately arranged in the *cis*- and *trans*-conformation, showed the relatively longer Ge-Sn bond distances (2.859 and 2.861 Å) than the sum of covalent radii of Ge and Sn (2.62 Å = 1.22 Å(*r*_Ge_) + 1.40 Å(*r*_Sn_)) [27]. Lastly, the Ge–Ge distances of the Ge_2_ dimer in two title compounds were nearly identical, 2.534 and 2.536 Å, and these values were also comparable to those in Eu_3.04(4)_Ca_5.96_In_8_Ge_7.77(2)_Sn_0.23_ and in the parental (Eu_1−*x*_Ca*_x_*)_3_In_2_Ge_3_ series [12].

As briefly mentioned earlier, the crystal structure of the (Eu_1−*x*_Ca*_x_*)_3_In(Ge_3−*y*_Sn_1+*y*_) phase can be compared with that of the (Eu_1−*x*_Ca*_x_*)_9_In_8_(Ge_1−*y*_Sn*_y_*)_8_ phase. Firstly, both phases contain the common structural moieties including the edge-sharing In(Ge/Sn)_4_ tetrahedra and the *cis-trans* Ge/In- or Ge/Sn-chains. However, the (Eu_1−*x*_Ca*_x_*)_9_In_8_(Ge_1−*y*_Sn*_y_*)_8_ phase exclusively includes the additional 1*D zig-zag* In-chains propagating along the *b*-axis and perpendicular to the *cis-trans* In/Ge-chains. In addition, the crystal structure contains two crystallographic mirror planes, respectively, along the (1/4 0 0) and (3/4 0 0) planes containing those *zig-zag* In-chains. Furthermore, the (Eu_1−*x*_Ca*_x_*)_3_In(Ge_3−*y*_Sn_1+*y*_)-type structure is very closely related to the previously reported the (Eu_1−*x*_Ca*_x_*)_4_In_3_Ge_4_ phase [12], in which the isotypic In*Tt*_4_ (*Tt* = Tetrels) tetrahedra with the Ge_2_ dimers (previously-called “dumbbells”) were connected directly to each other along the *a*-axis and via In atoms along the *c*-axis. Therefore, all these three phases can also be regarded as an intergrowth series with slightly different structural moieties.

**Figure 4:**
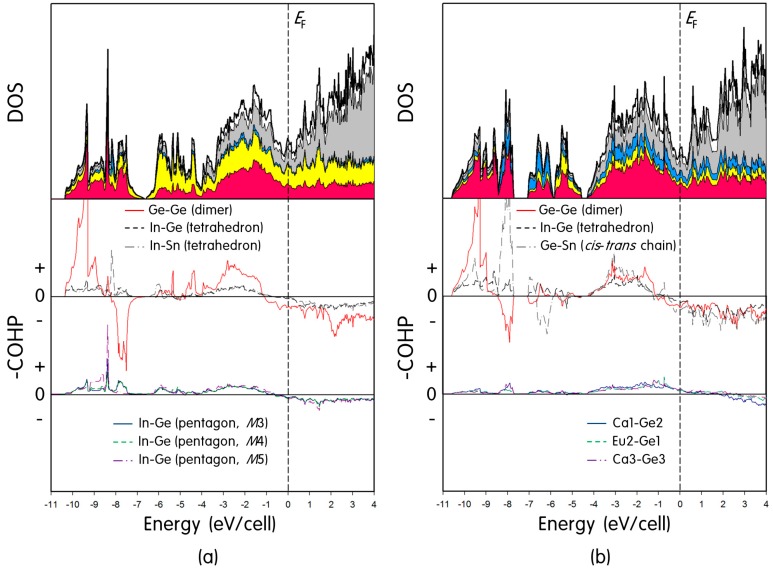
Density of states (DOS) and crystal orbital Hamilton population (COHP) curves for (**a**) “Eu_3_Ca_6_In_8_Ge_7_Sn” and (**b**) “EuCa_2_InGe_3_Sn”. Total and partial DOS curves are represented, respectively, by a solid-line and shaded areas with different colors as follows: Eu, white; Ca, gray; Sn, light blue; In, yellow; and Ge, magenta area. *E*_F_ (vertical line) is shown as a reference at 0 eV. Individual COHP curve is displayed: Ge-Ge forming the Ge_2_ dimer, In-Ge and In-Sn forming the In(Ge/Sn)_4_ tetrahedron, In-Ge forming different pentagons, Ge-Sn forming the *cis-trans* Ge/Sn-chain, and three cation-anion interactions. In the –COHP curves, the positive (“+”) values represent bonding interactions, whereas the negative (“–”) values represent antibonding interactions, respectively. The –COHP curves for each model are plotted in the same scale, respectively.

It is noteworthy to mentioned that the most noticeable difference between the (Eu_1−*x*_Ca*_x_*)_9_In_8_(Ge_1−*y*_Sn*_y_*)_8_ and the (Eu_1−*x*_Ca*_x_*)_3_In(Ge_3−*y*_Sn_1+*y*_) phases was the amount and the location of Sn-substitution: in the (Eu_1−*x*_Ca*_x_*)_9_In_8_(Ge_1−*y*_Sn*_y_*)_8_ phase, the 11% of Sn-substitution was successfully demonstrated only at one vertex of In(Ge/Sn)_4_ tetrahedron; whereas, in the (Eu_1−*x*_Ca*_x_*)_3_In(Ge_3−*y*_Sn_1+*y*_) phase, not only the partial Sn-substitution (*ca*. 13% or 27%) occurred at one vertex of the tetrahedron, but also the complete substitution happened at one Ge site on the *cis-trans* chain. Therefore, if the Zintl-Klemm concept is applied to this phase, the chemical formula can be re-written as [(Eu^2+^)_1__−_*_x_*(Ca^2+^)*_x_*]_3_[(In^−^)(Ge^−^)_3__−_*_y_*(Sn^−^)_1+*y*_]·(1e^−^), where a slightly charge-unbalanced formula should be expected. In addition, this chemical formula can explain the metallic property of the (Eu_1−*x*_Ca*_x_*)_3_In(Ge_3−*y*_Sn_1+*y*_) phase represented by the following DOS curves shown in Figure 4b.

### 2.2. Electronic Structure Calculations

A series of theoretical investigations have been systematically conducted using TB-LMTO method [24] to understand overall electronic structures of two title phases and chemical bonding among components [28,29,30]. Given the practical reason, a mixed-occupation of two atoms at one atomic site cannot be applied to computational calculations. Therefore, two structural models with idealized compositions of “Eu_3_Ca_6_In_8_–_7_Sn” and “EuCa_2_InGe_3_Sn”, respectively, representing the (Eu_1−*x*_Ca*_x_*)_9_In_8_(Ge_1−*y*_Sn*_y_*)_8_ and the (Eu_1−*x*_Ca*_x_*)_3_In(Ge_3−*y*_Sn_1+*y*_) phases were designed and exploited for our theoretical studies.

#### 2.2.1. Eu_3_Ca_6_In_8_Ge_7_Sn

To build a model with an idealized composition of Eu_3_Ca_6_In_8_Ge_7_Sn, firstly the experimentally obtained space group *Pmmn* was replaced by *Pmm*2 to divide one Ge1/Sn mixed-site (*Wyckoff* 4*f*) into two individual sites (*Wyckoff* 2*e* and 2*f*), and then Ge and Sn atoms were allocated to each site accordingly. In addition, two mixed-cationic sites having the larger Eu contents than other sites (*M*2 and *M*5) were solely assigned for Eu, whereas the rest of three mixed-cationic sites (*M*1, *M*3 and *M*4) were assigned for Ca. TB-LMTO calculations were executed using this structural model, and the resultant DOS and COHP curves are displayed in Figure 4a. Total and partial DOS curves show an overall valence orbital mixing of five components throughout the entire energy window. The local DOS minimum (so-called pseudogap) is observed at the Fermi level (*E*_F_) implying a semi-metallic property of this phase, just like its parental (Eu_1−*x*_Ca*_x_*)_9_In_8_Ge_8_ phase.

Total DOS curve can roughly be divided into three sectors below *E*_F_. The sector between −10.5 and −7 eV displays several large peaks, which represent Ge σ_s_ bonding- and σ_s_* antibonding-states originated from the bridging Ge_2_ dimer. The sector between −7 and −4 eV includes strong contributions descended from In 5*p* and Ge 4*p* states consisting of the 12-membered ring. Lastly, the sector from −4 to 0 eV shows significant contributions from In 5*p* and Ge 4*p* states with small additions from Sn 4*p* states, respectively, consisting of the In(Ge/Sn)_4_ tetrahedron and the Ge_2_ dimer. In particular, the noticeably large contributions from Eu and Ca are also observed in this sector implying that some degrees of bonding interactions between cations and anions should exist. This is one of the typical features of polar intermetallic compounds, which is caused by an incomplete electron transfer from cations to anions. Six COHP curves are also displayed in the middle and the bottom of Figure 4a. The Ge-Ge COHP curve from the Ge_2_ dimer shows a strong anti-bonding character at *E*_F_, whereas the In-Ge and the In-Sn COHP curves from the In(Ge/Sn)_4_ tetrahedron are optimized at *E*_F_. Three In-Ge COHPs representing various interactions on the 12-membered ring indicate relatively weak, but nearly optimized, interactions at *E*_F_.

#### 2.2.2. EuCa_2_InGe_3_Sn

For the structural model representing the (Eu_1−*x*_Ca*_x_*)_3_In(Ge_3−*y*_Sn_1+*y*_) phase, the orthorhombic space group *Pnma* was exploited as obtained from the SXRD refinement. However, the *M*2 site showing the largest Eu content among three mixed-cationic sites was solely assigned for Eu, and two other cationic sites were assigned for Ca. In addition, the mixed-site occupied by Ge3 and Sn2 was fully allocated by Ge to fulfill the idealized composition. It is noteworthy to mention that the Eu partial occupations over three mixed-cationic sites in the title phase resembled the partial occupation trend observed in the parental (Eu_1−*x*_Ca*_x_*)_3_In_2_Ge_3_ phase, where the largest Eu content was found at the *M*2 site, then the next largest one at the *M*1 site. The *M*3 site showed the smallest amount of Eu. The rationales for this site-preference of Eu were thoroughly investigated using both the coloring-problem and the QVAL value criteria in the earlier article [14].

A series of calculations was performed using EuCa_2_InGe_3_Sn, and DOS and COHP curves are illustrated in Figure 4b. The overall DOS curves and the location of *E*_F_ are similar to those obtained from Eu_3_Ca_6_In_8_Ge_7_Sn as compared in Figure 4. In addition, the total DOS curve below *E*_F_ can roughly be divided into three sectors as well. However, the specific orbital contributions to these sectors were slightly different from those in Eu_3_Ca_6_In_8_Ge_7_Sn. In particular, the sector between −10.5 and −7.5 eV is mostly contributed by Ge 4*s* and Sn 5*s* orbitals originated, respectively, from bonding- and antibonding-states of the Ge_2_ dimers, and bonding-states of the 1*D cis-trans* Ge/Sn chains. The sector between −7 and −4.5 eV has contributions from Ge 4*s*, Sn 5*s* and In 5*s* orbitals from antibonding-states of the infinite *cis-trans* Ge/Sn-chain and bonding-/antibonding-states of the In(Ge/Sn)_4_ tetrahedron, respectively. Lastly, the sector between −4 and 0 eV displays a strong orbital mixing of Ge 4*p*, In 5*p* and Sn 5*p* states with some contributions from two cations. Two COHP curves of the Ge-Ge and the Ge-Sn bonding, respectively, from the Ge_2_ dimer and the *cis-trans* Ge/Sn chain display small antibonding characters at *E*_F_, whereas the In-Ge COHP from the In(Ge/Sn)_4_ tetrahedron is nearly optimized at *E*_F_. Three COHP curves representing interatomic interactions between cations and anions (Ca1-Ge2, Eu2-Ge1, and Ca3-Ge3) show relatively weak and small bonding characters (bottom of Figure 4b). These favorable bonding interactions can compensate several unfavorable antibonding characters descended from the Ge-Ge and the Ge-Sn bonding and eventually stabilize the overall crystal structure in the given chemical composition.

**Figure 5:**
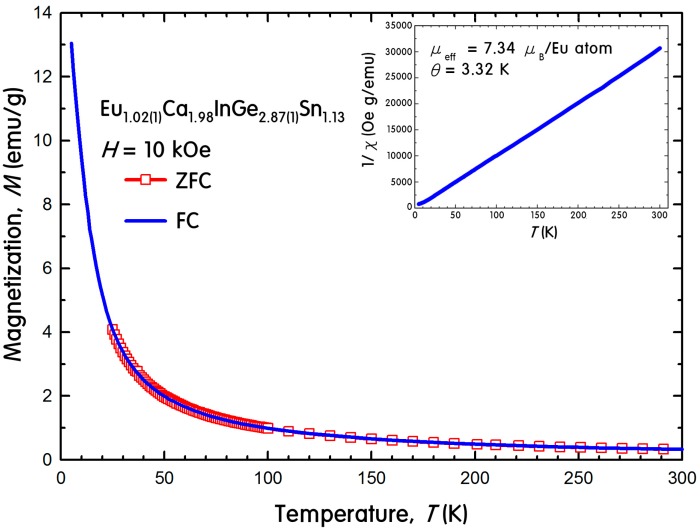
Magnetization of Eu_1.02(1)_Ca_1.98_InGe_2.87(1)_Sn_1.13_ as a function of temperature measured in dc magnetic field of 10 kOe under both ZFC and FC conditions. Inset shows the linear fit of temperature-dependent inverse magnetic susceptibility measured in 10 kOe under the FC condition.

### 2.3. Physical Property Measurements

The temperature-dependent dc magnetization was measured on a polycrystalline sample of Eu_1.02(1)_Ca_1.98_InGe_2.87(1)_Sn_1.13_ to study the magnetic interactions between Eu atoms. Since the magnetization study for the parental (Eu_1−*x*_Ca*_x_*)_3_In_2_Ge_3_ phase was unsuccessful due to the presence of a secondary phase in products, the current magnetic property for its quinary derivative is the first report for its kind. Figure 5 illustrates the magnetization as a function of temperature between 5 and 300 K under zero-field cooled (ZFC) and field cooled (FC) conditions using the dc magnetic field of 10 kOe. The inverse susceptibility is also shown as a function of temperature in inset.

**Figure 6:**
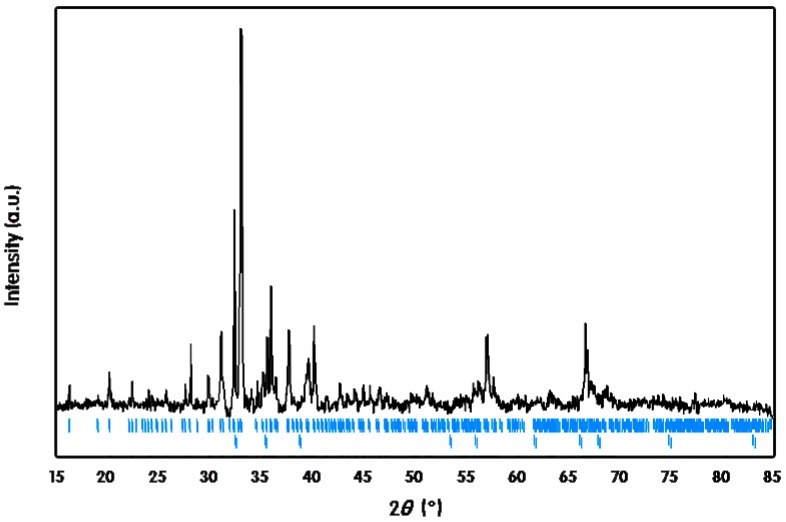
Powder X-ray diffraction (PXRD) pattern of Eu_1.02(1)_Ca_1.98_InGe_2.87(1)_Sn_1.13_. The upper and lower ticks shown in blue indicate the calculated reflection positions of Eu_1.02(1)_Ca_1.98_InGe_2.87(1)_Sn_1.13_ and elemental In, respectively.

**Figure 7:**
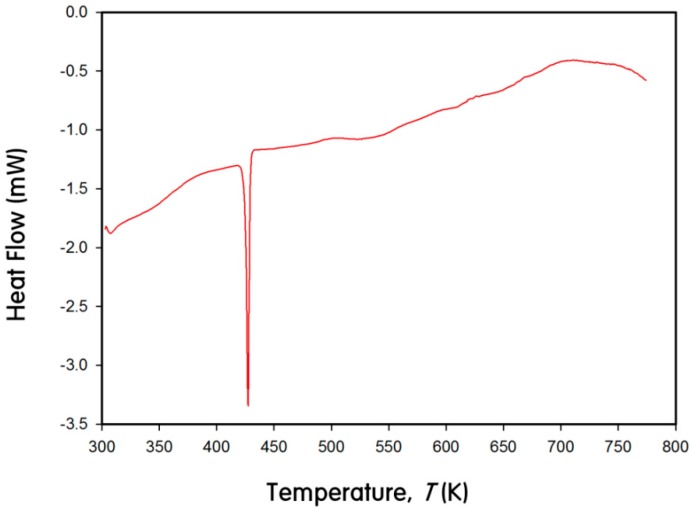
Differential scanning calorimeter (DSC) curve for Eu_1.02(1)_Ca_1.98_InGe_2.87(1)_Sn_1.13_ between 300 and 770 K.

Eu_1.02(1)_Ca_1.98_InGe_2.87(1)_Sn_1.13_ followed the Curie-Weiss law with the corresponding paramagnetic behavior, and there was no indication of magnetic ordering down to 5 K. The effective magnetic moment of 7.34 μ_B_ per Eu^2+^ ion was calculated from a linear fit of the inverse magnetic susceptibility *versus* temperature, and this value was relatively lower than the theoretically expected effective moment of 7.94 μ_B_ for a free Eu^2+^ ion. This kind of discrepancy between the experimental and the theoretical values was previously reported in our recent article about Eu_3.13(2)_Ca_5.87_In_8_Ge_8_, which was also synthesized by the molten In-metal flux method. The reason for this discrepancy was attributed to small amounts of remaining In metals even after the centrifugation conducted at the last stage of the flux reaction. The inclusion of In metals in our product was verified by powder X-ray diffraction (PXRD) (Figure 6), DSC (Figure 7) and SEM images analyses. The extrapolation of the linear fitting for the magnetic susceptibility curve in the paramagnetic region resulted in θ_P_ = +3.32 K, which indicated a weak and relatively low temperature ferromagnetic (FM) ordering of Eu atoms.

Figure 7 displays a DSC curve plotting the heat flow as a function of temperature change between 300 and 770 K. No exothermic or endothermic peak was observed except a peak at 420 K originated from In metal. This implies that there exists no impurity phase in a product and no possibility of structural transformation or decomposition of the product in the measured temperature range.

## 3. Experimental Section

### 3.1. Synthesis

All manipulations during the synthesis were carried out in an argon-filled glove-box with O_2_ and H_2_O contents of <0.1 ppm or under vacuum. The starting materials were used as purchased from Alfa, and the list of materials is as follows: Eu—ingot, 99.9%; Ca—shot, 99.5%; In—tear drop, 99.99%; Ge—pieces, 99.999%; and Sn—shot, 99.99%. Tanned surface of the Eu ingot was scrapped off using a scalpel before loaded in a reaction container. The molten In-metal flux reaction, in which excess amounts of In metals were used as a reactive flux, was carried out in an alumina crucible (*ca*. 2 cm^3^), and the loaded elemental ratios were Eu:Ca:Ge:Sn:In = 0.9:1.1:(1 − *x*):*x*:8 (*x* = 0.1–0.3) and 0.9:1.1:(2 − *x*):*x*:8 (*x* = 1.0–1.2), respectively, for derivatives of the (Eu_1−*x*_Ca*_x_*)_9_In_8_(Ge_1−*y*_Sn*_y_*)_8_ and the (Eu_1−*x*_Ca*_x_*)_3_In(Ge_3−*y*_Sn_1+*y*_) phases. After each elemental mixture was loaded in a reaction container, it was subsequently enclosed in a fused-silica ampoule by flame-sealing under vacuum. Then, the fused-silica ampoule was heated up to 960 °C at the rate of 200 °C/h by a box-type furnace, held there for 20 h, then cooled down to 500 °C at the rate of 5 °C/h. The extra amounts of molten In metals were removed by the instantaneous centrifugation at 500 °C. Large amounts of well-grown bundles of bar-/needle-shaped single-crystals with a silver luster were obtained from all three products. Eu_3.04(4)_Ca_5.96_In_8_Ge_7.77(2)_Sn_0.23_ was air-/moisture-sensitive and started to decompose after one day, whereas two compounds in the (Eu_1−*x*_Ca*_x_*)_3_In(Ge_3−*y*_Sn_1+*y*_) phase remained intact at least for one week.

### 3.2. Crystal Structure Determinations

Crystal structures of three title compounds have been characterized by both powder and single-crystal X-ray diffractions (SXRD). Powder X-ray diffraction (PXRD) patterns were obtained using Bruker D8 diffractometer (monochromatic Cu Kα_1_ radiation, λ = 1.54059 Å) with a step size of 0.05° in the range of 15° ≤ 2θ ≤ 85° and a total exposure time of 1 h. Primarily, a phase purity of each product was briefly checked, and lattice parameters of each unit cell were obtained using program *Rietica* [31]. Several peaks originated from remaining In metals were also indexed. SXRD data were collected using Bruker SMART APEX2 CCD-based diffractometer (Bruker, Billerica, MA, USA) equipped with Mo K*α*_1_ radiation (λ = 0.71073 Å) at room temperature. Firstly, several silvery lustrous bar-/needle-shaped single-crystals were isolated and selected from bundles or aggregates of each product. After then, the qualities of selected crystals were briefly checked by a rapid scan, and the best crystal was chosen for the further data collection. Full data collection was processed using the Bruker APEX2 software [32]. Data reduction, integration, and unit-cell refinements were carried out using SAINT program [33], and semi-empirical absorption correction based on equivalents was conducted using the SADABS program [34]. The program XPREP in the SHELXTL software package was exploited to sort and merge the structure factors [35]. Refined parameters included the scale factor, atomic positions with anisotropic displacement parameters, extinction coefficients, and occupation factors of five Eu/Ca mixed-sites and one Ge/Sn mixed-site for Eu_3.04(4)_Ca_5.96_In_8_Ge_7.77(2)_Sn_0.23_, and three Eu/Ca mixed-sites and one Ge/Sn mixed-site for Eu_0.95(1)_Ca_2.05_InGe_2.73(1)_Sn_1.27_ and Eu_1.02(1)_Ca_1.98_InGe_2.87(1)_Sn_1.13_, respectively.

During the structure refinement for two compounds in the (Eu_1−*x*_Ca*_x_*)_3_In(Ge_3−*y*_Sn_1+*y*_) series, we found that the atomic displacement parameters (ADP) for the Sn1 sites were slightly higher than those of Ge and In sites. These Sn1 sites were crystallographically the identical sites to the In2 sites of the parental (Eu/Ca)_3_In_2_Ge_3_ phase [12], and the ADP for the In2 sites in the parental phase were also slightly higher than those of other In- and Ge-sites. Therefore, the larger ADP at the given site should be regarded as a structural characteristic of this type of structure. However, in order to clarify any possibility of a partial occupation or a Ge-mixed occupation for the Sn1-sites, we attempted to refine the crystal structures by allowing free occupations at the given site. However, as the occupations of the Sn1-sites were set free, the values became 99.7(2)% and 100.3(3)%, respectively, for Eu_1.02_Ca_1.98_InGe_2.87_Sn_1.13_ and Eu_0.95_Ca_2.05_InGe_2.73_Sn_1.27_, and the ADP were kept nearly constant. Thus, we concluded that there was no further reason to consider either a partial or a mixed-occupation for the Sn1 sites.

In the last refinement cycle, atomic positions were standardized using STRUCTURE TIDY [36]. Important crystallographic data, atomic positions, thermal displacement parameters, and selected interatomic distances are listed in Table 1, Table 2 and Table 3. CIF Files are deposited in Fachinfor-mationszentrum Karlsruhe, 76344 Eggenstein-Leopold-shafen, Germany (Fax: (49) 7247-808-666; E-mail: crysdata@fiz.karlsruhe.de) with depository numbers of CSD-429251 for Eu_3.04(4)_Ca_5.96_In_8_Ge_7.77(2)_Sn_0.23_, CSD-429252 for Eu_1.02(1)_Ca_1.98_InGe_2.87(1)_Sn_1.13_ and CSD-429253 for Eu_0.95(1)_Ca_2.05_InGe_2.73(1)_Sn_1.27_.

**Table 3:** Selected interatomic distances (Å) for Eu_3.04(4)_Ca_5.96_In_8_Ge_7.77(2)_Sn_0.23_, Eu_1.02(1)_Ca_1.98_InGe_2.87(1)_Sn_1.13_ and Eu_0.95(1)_Ca_2.05_InGe_2.73(1)_Sn_1.27_.

Atomic Pair	Distance	Atomic Pair	Distance	
	Eu_3.04(4)_Ca_5.96_In_8_Ge_7.77(2)_Sn_0.23_		Eu_1.02(1)_Ca_1.98_InGe_2.87(1)_Sn_1.13_	Eu_0.95(1)_Ca_2.05_InGe_2.73(1)_Sn_1.27_
In1-Ge1/Sn(×2)	2.816(1)	In1-Ge2	2.779(1)	2.779(1)
In1-Ge1/Sn(×1)	2.896(2)	In1-Ge3/Sn2(×2)	2.811(1)	2.825(1)
In1-Ge2	2.770(1)	In1-Ge3/Sn2(×1)	2.898(1)	2.914(1)
In2-Ge1/Sn	3.270(1)	Sn1-Ge1	2.859(1)	2.861(1)
In2-Ge2	2.959(2)	Sn1-Ge1	2.947(1)	2.953(1)
In2-Ge3	2.981(2)	Sn1-Ge2	2.986(1)	2.987(1)
In2-Ge4	3.030(1)	Sn1-Ge3/Sn2	3.377(1)	3.377(1)
In3-Ge3	2.787(1)			
In3-Ge4	2.793(2)			
In3-In5	3.022(1)			
In4-Ge4	2.834(1)			
In4-In5	2.988(1)			
Ge2-Ge3	2.554(2)	Ge1-Ge2	2.536(1)	2.534(1)

### 3.3. Electronic Structure Calculations

Theoretical investigations have been carried out using two structural models with idealized compositions of “Eu_3_Ca_6_In_8_Ge_7_Sn” and “EuCa_2_InGe_3_Sn” representing for the (Eu_1−*x*_Ca*_x_*)_9_In_8_(Ge_1−*y*_Sn*_y_*)_8_ and the (Eu_1−*x*_Ca*_x_*)_3_In(Ge_3−*y*_Sn_1+*y*_) phases, respectively. The Stuttgart TB-LMTO47 program with the atomic sphere approximation (ASA) [25] was exploited, and exchange and correlation were treated by the local density approximation (LDA) [22]. All relativistic effects except spin-orbit coupling were taken into account by using a scalar relativistic approximation. In the ASA method, space is filled with overlapping Wigner-Seitz (WS) atomic spheres [25], and the symmetry of the potential is considered spherical inside each WS sphere. A combined correction is used to take into account the overlapping part [37]. The radii of WS spheres were determined by an automatic procedure and by requiring the overlapping potential be the best possible approximation to the full potential [37]. This overlap should not be too large because the error in kinetic energy introduced by the combined correction is proportional to the fourth power of the relative sphere overlap. No empty sphere [25] was necessary. The used WS radii for each model are listed as follows: Eu = 1.94–2.17 Å, Ca = 1.99–2.11 Å, In = 1.60–1.92 Å, Ge = 1.48–1.61 Å, and Sn = 1.66 Å for Eu_3_Ca_6_In_8_Ge_7_Sn; and Eu = 2.18 Å, Ca = 2.02–2.08 Å, In = 1.62 Å, Ge = 1.45–1.58 Å, and Sn = 1.81 Å for EuCa_2_InGe_3_Sn. The basis sets included 6*s*, 6*p* and 5*d* orbitals for Eu; 4*s*, 4*p* and 3*d* orbitals for Ca; 5*s*, 5*p* and 5*d* orbitals for In; 4*s*, 4*p* and 4*d* orbitals for Ge; and 5*s*, 5*p* and 5*d* orbitals for Sn for both models. The Eu 5*d*, Ca 3*d*, In 5*d*, and Ge 4*d* orbitals were treated by the Löwdin downfolding technique [25]. The 4*f* wave functions of Eu were treated as core functions. The *k*-space integrations were conducted by the tetrahedron method [38], and the self-consistent charge density was obtained using 360 and 216 irreducible *k*-points in the Brillouin zone, respectively, for Eu_3_Ca_6_In_8_Ge_7_Sn and EuCa_2_InGe_3_Sn.

### 3.4. EDS and SEM Images Analyses

Elemental analysis via EDS and product images of single-crystals were taken by ULTRA Plus field-emission SEM system (Zeiss, Oberkochen, Germany) with an acceleration voltage of 30 kV. Several bar-/needle-shaped single-crystals were selected from each product batch, and those crystals were carefully mounted on the circumference of an aluminum puck with double-sided conducting carbon tapes inside an argon-filled glove-box. EDS results are as follows: Eu_3.27(9)_Ca_5.68_In_8.49_Ge_7.80_Sn_0.24_, Eu_1.18(9)_Ca_1.84_In_1.45_Ge_2.75_Sn_0.80_, and Eu_0.98(9)_Ca_2.03_In_1.54_Ge_2.53_Sn_0.92_, and these values are comparable to the refined SXRD results as follows: Eu_3.04(4)_Ca_5.96_In_8_Ge_7.77(2)_Sn_0.23_, Eu_1.02(1)_Ca_1.98_InGe_2.87(1)_Sn_1.13_, and Eu_0.95(1)_Ca_2.05_InGe_2.73(1)_Sn_1.27_. Some deviations of In contents in both title phases can be attributed to the remaining In-flux metals stuck on samples.

### 3.5. DSC Measurement

Thermal characteristic of Eu_1.02(1)_Ca_1.98_InGe_2.87(1)_Sn_1.13_ was investigated by DSC using the TA instruments DSC2910 (TA Instruments, New Castle, DE, USA). The sample was enclosed in an aluminum container and heated from 300 up to 773 K at 10 K/min, then cooled down to 300 K at 10 K/min under N_2_ atmosphere.

### 3.6. Magnetic Property Measurements

Magnetization of Eu_1.02(1)_Ca_1.98_InGe_2.87(1)_Sn_1.13_ was measured by MPMS-7 using a polycrystalline sample weighing *ca*. 60 mg. To investigate the dc magnetization, the measurement was initially performed on heating the sample from 25 to 300 K under zero-field-cooled condition (ZFC). The measurement was repeated upon cooling from 300 to 5 K with a magnetic field of 10 kOe under field-cooled condition (FC).

## 4. Conclusions

Three Sn-substituted quinary polar intermetallic compounds in the (Eu_1−*x*_Ca*_x_*)_9_In_8_(Ge_1−*y*_Sn*_y_*)_8_ and the (Eu_1−*x*_Ca*_x_*)_3_In(Ge_3−*y*_Sn_1+*y*_) series have been synthesized using the molten In-metal flux method and characterized by both powder and single-crystal X-ray diffractions. Eu_3.04(4)_Ca_5.96_In_8_Ge_7.77(2)_Sn_0.23_ adopted its parental Eu_2.94(2)_Ca_6.06_In_8_Ge_8_-type structure, and the overall crystal structure was viewed as an assembly of the 3*D* polyanionic framework consisting of the 1*D* In(Ge/Sn)_4_ tetrahedral chain, the annulene-like 12-membered rings and the bridging Ge_2_ dimers, and the space filling mixed-Eu/Ca cations. The 12-membered ring can alternately be viewed as a combination of two different types of 1*D* anionic chain, the *zig-zag* and the *cis-trans*, propagating orthogonal directions to each other. Since the partial Sn-substitution for Ge was confined at the Ge1 site in the In(Ge/Sn)_4_ tetrahedron, the overall size of a unit cell as well as various interatomic distances were not much enlarged. Five mixed-cationic sites were locally surrounded by either 9 or 10 anions, respectively, forming a distorted pyramid or pentagonal-prisms. On the other hand, two derivatives in the (Eu_1−*x*_Ca*_x_*)_3_In(Ge_3−*y*_Sn_1+*y*_) phase were obtained as the loaded Sn content increased up to equal to or slightly higher than that of Ge. Unlike the parental phase, two derivatives were successfully crystallized in the given structure type even when the loaded Eu:Ca ratio was nearly 1:2. In addition, the overall atomic % of Eu included in compounds also increased up to 34 atomic %, which exceeded the previously claimed maximum limit of 20 atomic %. The rationale for this phenomenon should be attributed to the atomic size-factor: the Sn-substitution for Ge both in the In(Ge/Sn)_4_ tetrahedron and the 1*D cis-trans* chain caused polyanionic frameworks to expand resulting in accommodating extra amounts of larger-size Eu in compounds.

A series of theoretical investigations was conducted using two structural models of Eu_3_Ca_6_In_8_Ge_7_Sn and EuCa_2_InGe_3_Sn. The resultant DOS and COHP analyses indicated overall significant valence orbital mixings of all five components throughout the entire energy window and implied semi-metallic characters for both title phases. The temperature-dependent magnetization measurement for Eu_1.02(1)_Ca_1.98_InGe_2.87(1)_Sn_1.13_ proved the effective magnetic moment of 7.34 μ_B_ per Eu^2+^ ion and a low temperature FM ordering of Eu with θ_P_ = +3.32 K. The relatively low value of an effective magnetic moment was attributed to the presence of remaining In metals in a product. The DSC measurement also confirmed that there existed no secondary phase in Eu_1.02(1)_Ca_1.98_InGe_2.87(1)_Sn_1.13_ and no structural transformation or decomposition within the measured temperature range.
